# Ligand‐Dependent Intracluster Interactions in Electrochemical CO_2_ Reduction Using Cu_14_ Nanoclusters

**DOI:** 10.1002/smll.202409910

**Published:** 2024-12-04

**Authors:** Yamato Shingyouchi, Masaki Ogami, Sourav Biswas, Tomoya Tanaka, Maho Kamiyama, Kaoru Ikeda, Sakiat Hossain, Yusuke Yoshigoe, D. J. Osborn, Gregory F. Metha, Tokuhisa Kawawaki, Yuichi Negishi

**Affiliations:** ^1^ Department of Applied Chemistry Faculty of Science Tokyo University of Science 1−3 Kagurazaka, Shinjuku‐ku Tokyo 162–8601 Japan; ^2^ Research Institute for Science and Technology Tokyo University of Science 2641 Yamazaki Noda Chiba 278–8510 Japan; ^3^ Department of Chemistry University of Adelaide Adelaide South Australia 5005 Australia; ^4^ Carbon Value Research Center Tokyo University of Science 2641 Yamazaki Noda Chiba 278–8510 Japan; ^5^ Institute of Multidisciplinary Research for Advanced Materials Tohoku University Katahira 2‐1‐1, Aoba‐ku Sendai 980–8577 Japan

**Keywords:** CO_2_ reduction, CO_2_ reduction reaction, copper nanocluster, electrocatalyst, metal nanoclusters, nanoclusters

## Abstract

The electrochemical CO_2_ reduction reaction (CO_2_RR) has been extensively studied because it can be leveraged to directly convert CO_2_ into valuable hydrocarbons. Among the various catalysts, copper nanoclusters (Cu NCs) exhibit high selectivity and efficiency for producing CO_2_RR products owing to their unique geometric/electronic structures. However, the influence of protective ligands on the CO_2_RR performance of Cu NCs remains unclear. In this study, it is shown that different thiolate ligands, despite having nearly identical geometries, can substantially affect the electrochemical stability of Cu_14_ NCs in the CO_2_RR. Notably, Cu_14_ NCs protected by 2‐phenylethanethiolate exhibit greater stability and achieve a relatively higher selectivity (≈40%) for formic acid production compared with the cyclohexanethiolate‐protected counterpart. These insights are crucial for designing Cu NCs that are both stable and highly selective, enhancing their efficacy for electrochemical CO_2_ reduction.

## Graphical abstract

This study provides insight into the precise design of Cu nanocluster catalysts for electrochemical CO_2_ reduction reactions through atomic‐scale ligand engineering, using two similar ligands (i.e., CHT and PET). Cu_14_–CHT and Cu_14_–PET nanoclusters exhibit significant differences in selectivity and stability, despite having nearly identical geometric and electronic structures.

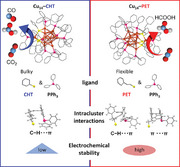

## 1 Introduction

In recent years, the electrochemical CO_2_ reduction reaction (CO_2_RR) driven by electricity derived from renewable energy sources has garnered significant attention as a promising strategy to achieve carbon neutrality.^[^ ^1^ ^]^ This process involves the direct conversion of CO_2_ into valuable hydrocarbon compounds under ambient temperature and pressure conditions. Copper (Cu) has been extensively studied in this context because it uniquely facilitates the production of various CO_2_RR products.^[^ ^1a^ ^]^ Notably, Cu nanoclusters (NCs) exhibit distinct electronic and geometric structures compared with larger Cu nanoparticles (NPs) and bulk Cu, primarily owing to quantum size effects associated with their ultrafine particle size.^[^ ^2^ ^]^ Thus, it is possible to modulate their electronic and geometric configurations by controlling the number of constituent atoms, which can be exploited to optimize the selectivity and efficiency of CO_2_RR products.^[^ ^3^ ^]^ Consequently, there has been a surge in research focused on the electrochemical CO_2_ reduction capabilities of Cu NCs.^[^ ^4^ ^]^

In catalytic applications, the organic ligands that protect the metal NC surface often play a crucial role in their activity.^[^ ^5^ ^]^ Particularly, in electrochemical catalysis, several effects have been observed. Notably, 1) decreasing the length of alkyl chains on the ligands can increase the activity of various electrocatalytic reactions by reducing the charge resistance between the electrode and the metal NCs,^[^ ^6^ ^]^ and 2) the introduction of hydrophilic functional groups can enhance the electrocatalytic activity of the hydrogen evolution reaction (HER) by facilitating the conduction of protons as reactants.^[^ ^7^ ^]^ Additionally, several studies have considered the effects of ligands on electrochemical CO_2_RRs using metal NCs. For example, 1) the selectivity and activity of CO_2_RRs can change depending on the element directly coordinated to the metal core in the ligand.^[^ ^8^ ^]^ Moreover, 2) hydrides within the ligands can impact the activity of the NCs,^[^ ^9^ ^]^ and 3) partial desorption of ligands can promote catalytic activity.^[^ ^10^ ^]^ These findings underscore the importance of ligand design in optimizing the performance of metal NCs in various catalytic processes.

However, most studies have focused on relatively stable noble metal NCs, such as gold (Au) and silver (Ag).^[^ ^8^, ^10^, ^11^, ^12^ ^]^ Few reports compare the effects of different ligands on electrochemical CO_2_RRs using Cu NCs, which are considered base metal NCs.^[^ ^4f^ ^]^ This is because, unlike Au and Ag NCs, Cu NCs are more prone to valence changes because of their lower ionization energy. Consequently, minor modifications in the ligand framework or the reduction conditions can significantly alter the number of constituent atoms and the geometric structure of Cu NCs.^[^ ^3^, ^13^ ^]^ This sensitivity complicates the study of ligand effects on the electrocatalytic behavior of Cu NCs, leading to a less comprehensive understanding compared to Au and Ag NCs.

**Figure 1:**
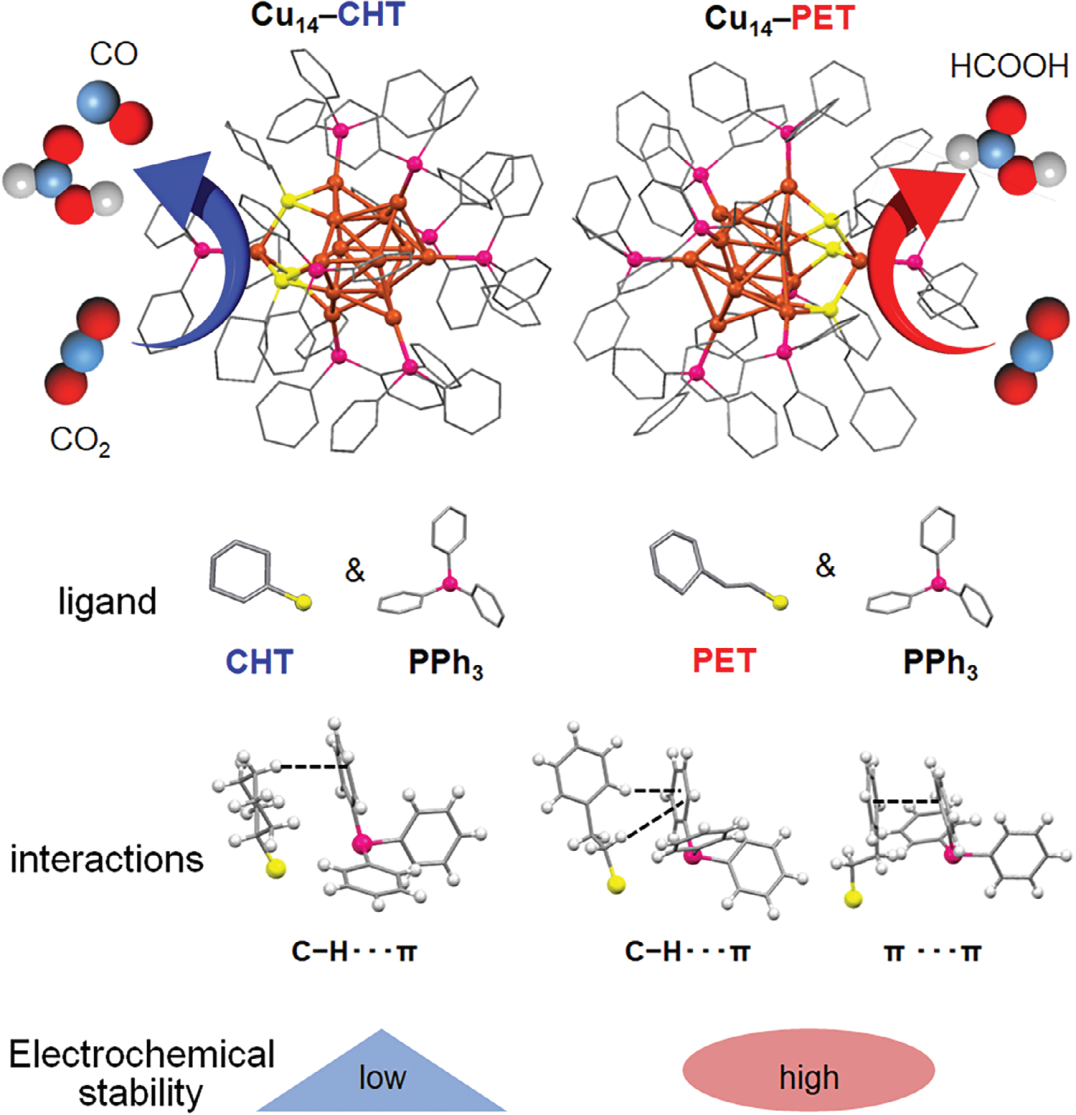
Schematic illustration of the two NCs investigated in this study. The two SR‐protected Cu_14_ NCs exhibit different interactions and reaction selectivity for electrochemical CO_2_ reduction reaction.

In this study, we synthesized Cu_14_ NCs with nearly identical geometric structures, coprotected by two different types of organic ligands: a thiolate (SR) and a phosphine. Furthermore, we used and compared two different SR ligands, namely 2‐phenylethanethiolate (PET) and cyclohexanethiolate (CHT), to stabilize the Cu_14_ NCs (Cu_14_–CHT and Cu_14_–PET) and investigated their effects on the electrochemical CO_2_RR (**Figure** **1**). Our findings revealed that subtle differences in the intracluster *π*···*π* and C─H···*π* interactions among the surface‐protected ligands can significantly impact the electrochemical stability of these NCs. In addition, this variation in SR ligands affects the metallophilic interactions, ultimately influencing the overall size of the NC's structural architecture, which may influence the stability of the intermediates formed during electrochemical CO_2_RRs. As a result, the small difference in SR ligands led to a significant variation in the sustained selectivity of formic acid (HCOOH) as a CO_2_RR product. The Cu NCs catalysts with such enhanced HCOOH selectivity has extremely unique properties not found in conventional monometallic bulk Cu or Cu NPs catalysts.^[^ ^2^ ^]^ Such a relatively high selectivity toward HCOOH might be due to the electronic state of Cu NCs that makes the formation of the HCOO^*^ intermediate more favorable than the ^*^COOH intermediate.^[^ ^4b,f,g^ ^]^

## 2 Result and Discussion

### 2.1 Synthesis and Characterization of Cu_14_ NCs

**Figure 2:**
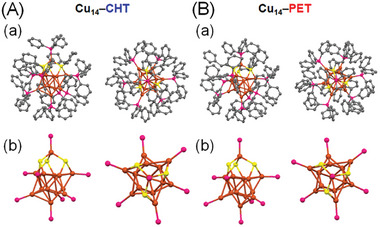
Cu_14_ frameworks of A) Cu_14_–CHT and B) Cu_14_–PET, showing the a) total geometric structure and b) core structure from the top and side views (orange =  Cu, yellow  =  S, magenta  =  P, and dark gray  =  C). Hydrogen atoms are removed for the clarity.

We synthesized Cu_14_ NCs coprotected by SR ligands and PPh_3_, using a synthesis process adapted from a previously reported method with slight modifications (Schemes S1, S2, Supporting Information).^[^ ^14^ ^]^ Two different SR ligands were strategically chosen for comparison: PET, which has a relatively flexible framework with a primary carbon at the *α*‐position and a phenyl (Ph) group, and CHT, which has a relatively rigid framework without a Ph group. The coprotected Cu NC, [Cu_14_(SR)_3_(PPh_3_)_8_H_10_]^+^, contains a distorted *fcc* structure with an outer Cu_8_ cube and an inner octahedral Cu_6_ core (**Figure** **2**; Figure S1, Supporting Information). When the bidentate SR (*o*‐carborane‐1,2‐dithiolate; C_2_B_10_H_10_S_2_) ligand is used, the regular *fcc* structure of the [Cu_14_(C_2_B_10_H_10_S_2_)_6_(CH_3_CN)_8_] NC with acetonitrile coordinated at the cubic vertex is obtained (Figure S1, Supporting Information).^[^ ^15^ ^]^ However, replacing the bidentate SR with the monodentate SR and PPh_3_ leads to structural distortion due to stretching of the outer Cu_8_ cubic framework of the [Cu_14_(SR)_3_(PPh_3_)_8_H_10_]^+^ NC structure.^[^ ^14^ ^]^ This distortion is more prominent when one Cu atom protrudes from the NC through SR bridging (Figure 2; Figure S1, Supporting Information), and the distorted structure can be stabilized by various intracluster interactions between the surface‐protected ligands.^[^ ^16^ ^]^ However, the presence of bulkier SR ligands introduces steric hindrance, which plays a significant role in enhancing such distortion by preventing the attachment of one PPh₃ unit into the NC, resulting in the formation of [Cu_14_(*^t^* BuS)_3_(PPh_3_)_7_H_10_]^+^.^[^ ^16a^ ^]^ Zhu et al. described a ligand exchange technique to generate a relatively stable variant of the [Cu_14_(SR)_3_(PPh_3_)_8_H_10_]^+^ NC, originating from [Cu_41_(SC_6_H_3_F_2_)_15_Cl_3_(P(PhF)_3_)_6_(H)_25_]^2−^ NCs, through the introduction of PPh_3_ ligands (Figure S2, Supporting Information).^[^ ^17^ ^]^

**Figure 3:**
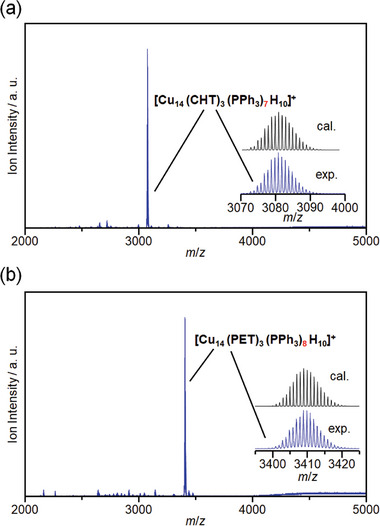
Positive‐ion ESI mass spectrum of a) Cu_14_–CHT and b) Cu_14_–PET. Insets show a comparison between the isotope patterns obtained from the experimental and calculated spectra.

Overall, [Cu_14_(SR)_3_(PPh_3_)_8_H_10_]^+^ NCs are relatively stable despite some structural distortion, which is limited but can be further increased by incorporating bulkier SR ligands. Given this constraint, we selected two different SR ligands that maintain an identical final molecular formula, namely [Cu_14_(CHT)_3_(PPh_3_)_8_H_10_]^+^ (Cu_14_–CHT) and [Cu_14_(PET)_3_(PPh_3_)_8_H_10_]^+^ (Cu_14_–PET). Single‐crystal X‐ray diffraction (SC‐XRD) results reveal that both NCs are crystallized in a similar triclinic crystal system with a similar *P*‐1 space group (Figure 2; Figure S3 and Tables S1, S2, Supporting Information). Although the basic framework structure of these two NCs is similar, the change in the SR ligand results in distinct Cu─Cu and Cu─S interactions, leading to a reduction in the overall size of the Cu_14_–PET NC architecture (Figure S4, Supporting Information). In Cu_14_–CHT NCs, we observed a slightly higher average Cu─Cu distance (2.5856 ± 0.0174 Å) and Cu─S distance (2.3178 ± 0.0191 Å) compared with those in Cu_14_–PET NC (2.5499 ± 0.0239 and 2.3048 ± 0.0145 Å, respectively). This causes a noticeable size difference between the two NCs, with the longest distance between two opposite Cu atoms being 6.861 Å in Cu_14_–CHT NC and 6.751 Å in Cu_14_–PET NC. Thus, it is expected that the intracluster interactions are enhanced in Cu_14_–PET NC. Additionally, in Cu_14_–CHT NC, we observed prominent intracluster C─H···*π* interactions, with a minimum distance of ≈3.2928 Å. In Cu_14_–PET NC, we identified two different types of C─H···*π* interactions: alkyl C─H···*π* interactions with the π orbitals of PPh_3_ units (≈3.0876 Å) and Ph C─H···*π* interactions with the π orbitals of PPh_3_ units (≈3.5894 Å). Furthermore, a T‐shaped *π*···*π* interaction between Ph and the PPh_3_ unit was also observed at a distance of ≈3.9285 Å. Therefore, from the combination of these intracluster interactions, the Cu_14_–PET structure is expected to be more stable compared with the Cu_14_–CHT structure. X‐ray photoelectron spectroscopy (XPS) confirmed that there is no difference in the charge state of Cu and SR in the two Cu_14_ NCs (Figure S5, Supporting Information). However, while we have identified the attachment of SR and PPh_3_ ligands to the Cu atoms through SC‐XRD measurements, the presence of hydrides cannot be accurately detected by SC‐XRD. Therefore, we confirmed the presence of hydrides by employing electrospray ionization mass spectrometry (ESI‐MS) (**Figure** **3**). Although the obtained molecular peaks indicate the presence of 10 hydrides in each NC, distinct fragmentation patterns are observed, correlating with their differences in stability. The single molecular ion peak obtained for Cu_14_–PET NC at *m*/*z* = 3409 suggests its higher stability, whereas dissociated peaks of the PPh_3_‐ligand (i.e., [Cu_14_(CHT)_3_(PPh_3_)_7_H_10_]^+^) were observed for Cu_14_–CHT NC at *m*/*z* = 3081. The isotropic distribution of all peaks confirms their mono‐cationic nature and matches with the theoretical pattern, verifying their molecular formula.^[^ ^14^ ^]^

### 2.2 Characterization of Cu_14_ NCs Loaded on Carbon Black

**Figure 4:**
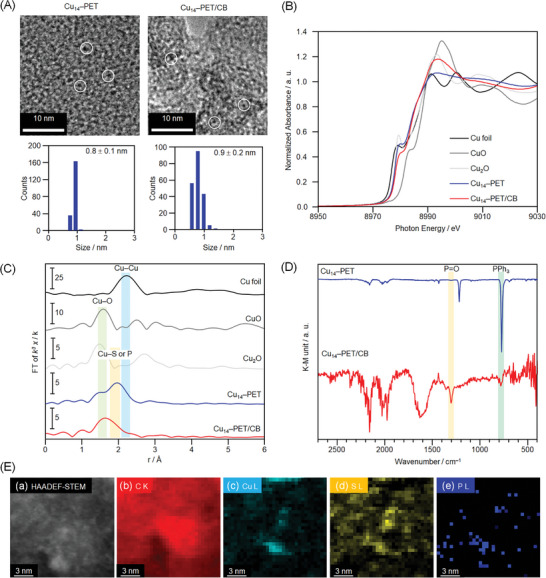
Characterization of Cu_14_–PET before and after deposition on CB (Cu_14_–PET/CB). A) TEM images and resulting histograms of the particle‐size distributions, Cu K‐edge B) XANES spectra, C) FT‐EXAFS spectra, and D) FT‐IR spectra. E) HAADF‐STEM images and EELS elemental mapping of Cu_14_–PET/CB (C–K, Cu–L, S–L═, and P–L). In B,C), Cu K‐edge XANES and FT‐EXAFS spectra of the Cu foil, CuO powder, and Cu_2_O powder are also shown for comparison. In C), the peaks at ≈1.6, ≈1.8, and 2.0–2.6 Å are assigned to the Cu─C or Cu─O, Cu─S or Cu─P, and Cu─Cu bonds, respectively. In B,C), the sample of Cu_14_–PET NC was measured at 10 K.

To evaluate the electrochemical CO_2_RR activity, we prepared the catalyst by loading the Cu_14_ NCs onto carbon black (CB) using the impregnation method (Scheme S3, Supporting Information). In the unit cell of each Cu_14_ NC, there are two NC units positioned in opposing directions to minimize intercluster interactions, ensuring their discrete nature in the solution medium (Figure S6, Supporting Information). However, upon loading onto CB, no significant change in their discrete nature is observed by transmission electron microscopy (TEM) (**Figure** **4**A; Figure S7, Supporting Information). Furthermore, the stability of the NCs upon loading on the CB surface is confirmed by X‐ray absorption fine structure (XAFS) measurements (Figure 4B,C; Figures S8, S9, Supporting Information). In the Cu K‐edge X‐ray absorption near‐edge structure (XANES) spectra, the pre‐edge peak (≈8980 eV) is known to vary significantly in shape depending on its valence and coordination environment, but both NCs showed no significant change for the pre‐edge peak position, confirming the consistent electronic state of Cu(I) before and after loading onto CB (Figure 4B; Figure S8a, Supporting Information). However, the Cu K‐edge Fourier transform‐extended XAFS (FT‐EXAFS) spectra reveal a relatively weaker Cu─P or Cu─S bond after loading onto the CB surface (Figure 4C; Figures S8b, S9, Supporting Information). This indicates the desorption of some ligands during the loading process, while the overall electronic structure of Cu(I) is maintained. Therefore, a slight structural change in the Cu_14_ NCs is likely caused by the desorption of neutral PPh_3_ ligands, not anionic SR. This is further supported by FT‐infrared spectroscopy (FT‐IR) (Figure 4D; Figure S10, Supporting Information) and XPS spectra (Figure S5, Supporting Information). Specifically, the appearance of the P═O stretching vibration at ≈1300 cm^−1^ in both Cu_14_ NCs after loading onto CB indicates the interaction of the neutral ligands with the CB surface, ultimately resulting in their desorption from the NC through the formation of O═PPh_3_.^[^ ^18^ ^]^ XPS spectra reveal similar binding energies for Cu and S before and after loading both NCs onto CB, further confirming their stability (Figure S5, Supporting Information). Finally, high‐angle annular dark‐field scanning TEM (HAADF‐STEM) and electron energy‐loss spectroscopy (EELS) confirm the presence of the constituent elements on the catalyst surface (Figure 4E).

### 2.3 CO_2_RR Activity of Cu_14_ NCs Loaded on CB

**Figure 5:**
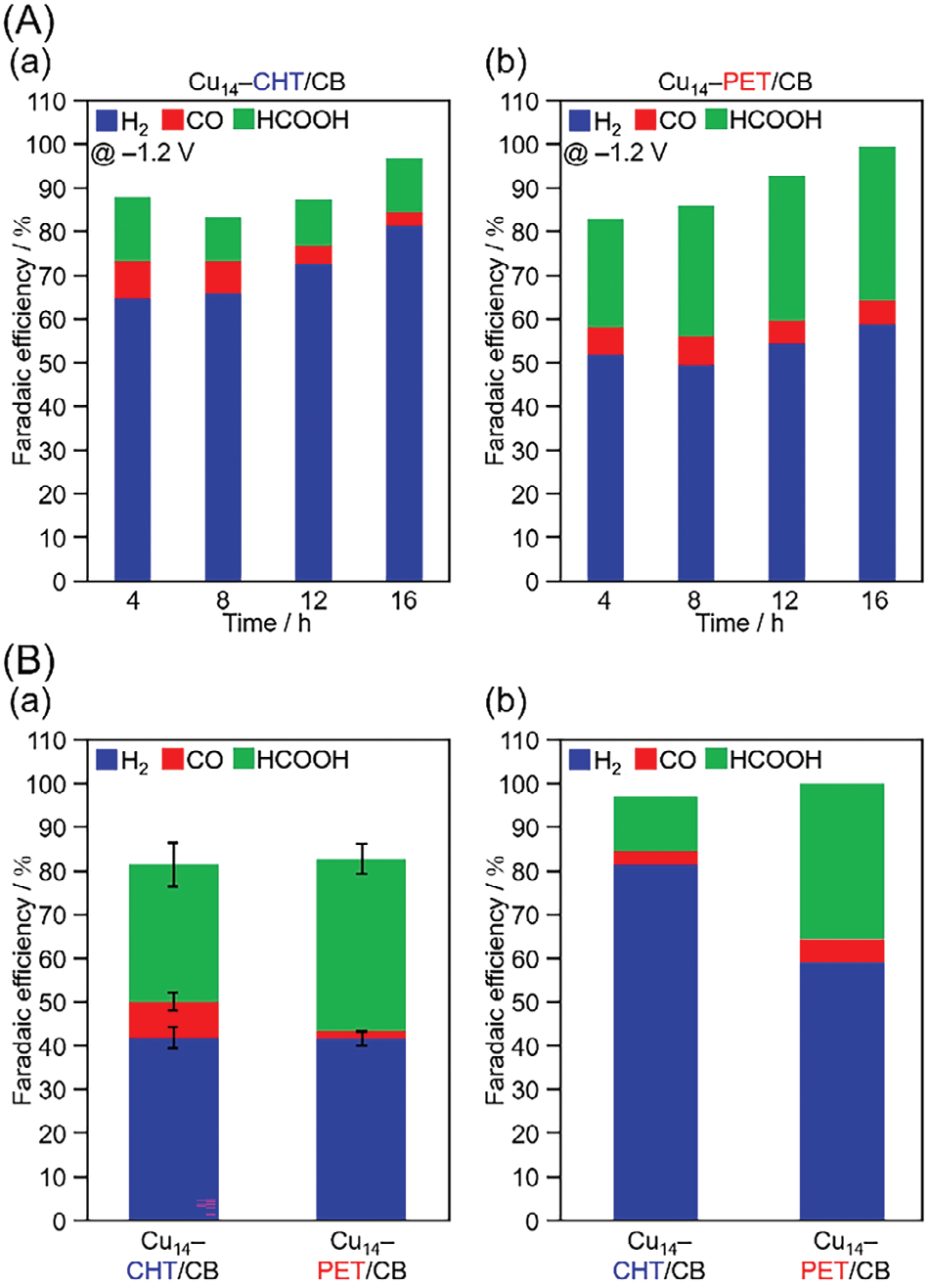
A) Time dependence of the FE for the CO_2_ reduction products of a) Cu_14_–CHT/CB and b) Cu_14_–PET/CB and B) the resulting FE for the a) initial products (after 2 h) and the b) long‐term products (after 16 h) of Cu_14_–CHT/CB and Cu_14_–PET/CB.

Electrochemical measurements of Cu_14_ NCs loaded on CB were performed following the protocol shown in Scheme S4 (Supporting Information). **Figure** **5** displays the gas chromatography and ^1^H NMR determinations of the gas and liquid products, confirming the production of HCOOH and CO (Figure S11, Supporting Information). To verify that these products were derived from flowing CO_2_, constant‐potential electrolysis was performed in an Ar atmosphere, instead of CO_2_. The absence of any CO_2_RR products at all tested potentials confirmed that the products were generated from CO_2_ and not from another source (Figures S12, S13, Supporting Information).^[^ ^19^ ^]^

The Faraday efficiency (FE) data shown in Figure 5 and Figure S14 (Supporting Information) indicate that HCOOH is the predominant high‐value CO_2_RR product formed in both cases throughout the experimental potential range from −0.8 to −1.4 V versus reversible hydrogen electrode (RHE). For Cu_14_–CHT/CB, we found that the FE of HCOOH and CO (FE_HCOOH_ and FE_CO_) were ≈31% and ≈8% at −1.2 V versus RHE, respectively, indicating the competitive formation of these products. Furthermore, the competing reaction, HER, was also relatively likely to occur. In contrast, for Cu_14_–PET/CB, FE_HCOOH_ and FE_CO_ were ≈39% and ≈2% at the same potential, demonstrating a higher selectivity for HCOOH formation. Furthermore, the partial current density for HCOOH (*j*_HCOOH_), illustrated in Figure S14 (Supporting Information), indicates that Cu_14_–PET/CB exhibited a higher *j*_HCOOH_ compared with Cu_14_–CHT/CB at every tested potential (Figure S14, Supporting Information). This suggests that Cu_14_–PET/CB is more efficient in producing HCOOH, reinforcing its higher selectivity for the high‐value CO_2_RR product.

Moreover, the stability of the two Cu_14_ NCs for CO_2_RR was evaluated by conducting long‐term chronoamperometric measurements at −1.2 V versus RHE (Figure 5). The results indicate that the selectivity for HCOOH production gradually decreased in Cu_14_–CHT/CB over time, whereas Cu_14_–PET/CB maintained stable production of HCOOH. After 16 h, the FE_HCOOH_ was ≈12% for Cu_14_–CHT/CB and ≈35% for Cu_14_–PET/CB, demonstrating a significant difference in selectivity between the two catalysts. For Cu_14_–CHT/CB, the formation of HCOOH decreased over time, whereas the production of H_2_ (FE_H2_ of ≈58% at 2 h and ≈81% at 16 h) increased. In contrast, Cu_14_–PET/CB showed consistent FE_HCOOH_ values, indicating its superior stability and continuous selectivity for HCOOH production under prolonged electrochemical conditions.

### 2.4 Origin of the Difference in HCOOH Selectivity

Compared with other Cu NCs that show high selectivity for HCOOH, ^[^ ^4a,b^ ^]^ Cu_14_–SR is presumed to be relatively prone to H^*^ adsorption as well as the formation of the HCOO^*^ intermediate. Here, as previously discussed, both Cu_14_ NCs possess identical geometric and electronic structures. However, we observed a notable difference in their electrochemical CO_2_RR efficiency and product selectivity. We assume that this discrepancy arises from slight differences in the SR ligand structures, which affect the size and stability of the NCs.

Specifically, the differences in SR ligand structures likely influence the interaction of the NCs with CO_2_ molecules during the electrochemical process. These structural variations may alter the number of adsorption sites available for CO_2_, thereby impacting the overall efficiency and selectivity of the CO_2_RR. Moreover, the size and stability of the NCs are crucial factors, as they directly influence the durability and performance of the catalysts under prolonged electrochemical conditions. We observed that even minor variations in ligand structures can significantly impact the overall catalytic properties of these NCs. According to the SC‐XRD results, Cu_14_–PET exhibits stronger cuprophilic interactions, which reduce the overall size of the cluster and effectively enhance the intracluster *π*···*π* and C─H···*π* interactions between the surface‐protecting ligands (Figures S4, S15, Supporting Information). This also indicates that Cu_14_–PET is more stable than Cu_14_–CHT. During catalyst preparation, we observed that some neutral ligands desorbed from the structure based on the results of FT‐IR and XAFS analysis (Figures 4B–D). Therefore, we assume that CO_2_ specifically adsorbs onto vacant sites created by the removal of these neutral ligands, initiating the electrochemical reduction reaction. In particular, it is assumed that the reaction proceeds mainly at the Cu sites where the PPh_3_ in the specific Cu(SR)_3_(PPh_3_) motif has been removed. Furthermore, the stability of intermediates and the accessibility of active catalytic sites dictate product selectivity, which varies between the two catalysts. Cu_14_–PET, with its higher cuprophilic interaction and smaller size (Figure S4, Supporting Information; 6.861 Å vs. 6.751 Å), provides greater stability for intermediates, favoring the production of HCOOH over CO. Such a relatively high selectivity toward HCOOH might be due to the electronic state of Cu_14_–PET that makes the formation of the HCOO^*^ intermediate more favorable than the ^*^COOH intermediate.^[^ ^4b,f,g^ ^]^ In contrast, Cu_14_–CHT produces more CO during the early stages of electrochemical reduction. This is assumed that ^*^COOH intermediate was more easily formed in Cu_14_–CHT. However, after prolonged electrochemical exposure, we observed a shift in product selectivity. We attribute this change to the desorption of ligands, which exposes more catalytic sites and increases competition among the products. Generally, in CO_2_ reduction with fine Cu NPs, low‐coordinated Cu sites are preferred for the HER and CO formation, which reduce the selectivity for high‐value products.^[^ ^2^, ^20^ ^]^ We observed a similar trend: during prolonged electrochemical reduction, Cu_14_–CHT exhibited increases in the HER formation, whereas Cu_14_–PET maintained the HER and CO production and enhanced the formation of HCOOH. This indicates that both types of NCs experience the desorption of ligands during extended electrochemical exposure, with Cu_14_–CHT NC showing more pronounced ligand desorption.

**Figure 6:**
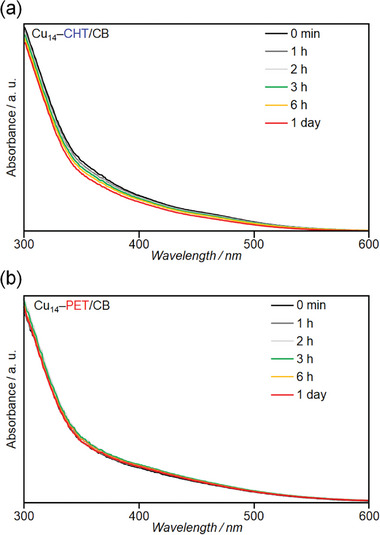
Time dependence of the DR spectra for a) Cu_14_–CHT and b) Cu_14_–PET in air.

However, tracking the exact number of desorbed ligands during the electrochemical process is challenging. Instead, we can infer the desorption pathway by correlating their behavior observed during ESI‐MS measurements. As previously discussed, both NCs exhibit different molecular ion peaks, despite being subjected to the same ionization conditions (Figure 3). More specifically, Cu_14_–PET exhibited the highest stability, as evidenced by a single molecular ion peak at *m*/*z* = 3409, confirming that all ligands remained intact. In contrast, Cu_14_–CHT demonstrated much lower stability, with fragmentation into different molecular ion peaks due to the desorption of its weakly coordinated neutral ligands. We identified the main peak at *m*/*z* = 3081 that corresponds to [Cu_14_(CHT)_3_(PPh_3_)_7_H_10_]^+^. Thus, the desorption of neutral ligands is more difficult in Cu_14_–PET owing to the prominent intracluster *π*···*π* and C─H···*π* interactions, compared with the loosely interacted Cu_14_–CHT. To further investigate the ease of PPh_3_ desorption, we investigated the stability of Cu_14_ NCs to air oxidation in the solid state, where degradation is likely to occur. As expected, the diffuse reflectance (DR) spectra of Cu_14_–CHT changed significantly with time compared with that of Cu_14_–PET, indicating relatively rapid degradation by oxygen in air (**Figure** **6**). This result also shows that the chemical stability of the Cu_14_ NCs differs significantly depending on the ease of PPh_3_ desorption, suggesting that PPh_3_ desorbs more easily in Cu_14_–CHT than in Cu_14_–PET.^[^ ^14^ ^]^

**Figure 7:**
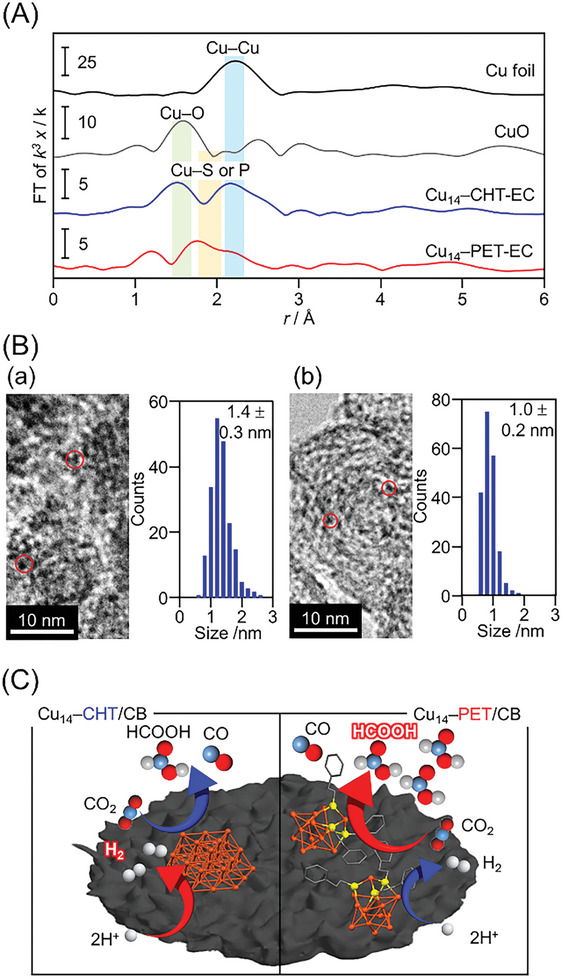
Characterization of Cu_14_–SR (SR = CHT and PET)‐loaded CB catalysts after the relatively prolonged electrocatalytic CO_2_RR (6 h; Cu_14_–SR/CB‐EC). A) Cu K‐edge FT‐EXAFS spectra of Cu_14_–SR/CB‐EC and B) TEM images with histograms of the particle‐size distributions for a) Cu_14_–CHT/CB‐EC and b) Cu_14_–PET/CB‐EC. C) Schematic of Cu_14_–SR/CB during long‐term electrocatalytic CO_2_ reduction. In A), Cu K‐edge XANES spectra of the Cu foil, and CuO powder are also shown for comparison, and the peaks at ≈1.6, ≈1.8, and 2.0–2.6 Å are assigned to the Cu─C or Cu─O, Cu─S or Cu─P, and Cu─Cu bonds, respectively. In C), in long‐term activity evaluation, H_2_ production becomes predominant in Cu_14_–CHT/CB, unlike Cu_14_–PET/CB. This change in product is presumed to be due to the promotion of Cu_14_–CHT aggregation during the catalytic reaction, which changes the Cu catalyst into one favorable for H_2_ production.

Although the desorption of neutral ligands during the electrochemical reduction process influences the performance, the stability of the overall electronic and geometric structure also plays a crucial role, significantly affecting product selectivity in CO_2_RR. Therefore, the structures of the catalysts were further evaluated after the relatively prolonged electrochemical CO_2_RR (6 h) (**Figure** **7**; Figures S16–S18, Supporting Information). The Cu K‐edge FT‐EXAFS spectra in Figures 4C and 7A show that Cu_14_–PET/CB maintains a similar spectral feature before and after the reaction, indicating intact Cu─P or Cu─S bonds. In contrast, the Cu─P or Cu─S bond (≈2.2 Å) disappeared and the intensity of the Cu─Cu (≈1.9 Å) bond increased for Cu_14_–CHT/CB after the reaction (Figure 7A; Figure S8, Supporting Information). However, similar pre‐edge peaks are observed in the Cu K‐edge XANES spectra, confirming the monovalent nature of the Cu atoms, even after the electrochemical reduction. In addition, the FT‐IR spectrum suggests the reductive desorption of O═PPh_3_ from the catalyst surface during electrochemical measurements (Figure S18, Supporting Information). We assume that this is a crucial reason why the total FE of all products (FE_Total_) does not reach 100%, considering that some of the electrons may be consumed in this reaction. In addition, a peak mainly attributed to PPh_3_ (≈800 cm^−1^) was observed in Cu_14_–PET/CB after the reaction, whereas this peak was not observed for Cu_14_–CHT/CB.^[^ ^21^ ^]^ Thus, PPh_3_ was relatively stable in Cu_14_–PET/CB than Cu_14_–CHT/CB during the reaction, but PPh_3_ was almost entirely removed from Cu_14_–CHT/CB. Furthermore, XPS measurements indicated the presence of SR ligands in the Cu_14_–PET/CB catalyst after the reaction, whereas SR ligands were absent in the Cu_14_–CHT/CB catalyst (Figure S16, Supporting Information). Therefore, although Cu(I) atoms persist, the overall structural integrity of Cu_14_–CHT NC is compromised during electrochemical reduction, which begins with the desorption of neutral ligands.

Finally, TEM images of the catalysts after electrochemical reduction were obtained to investigate the change in particle size (Figure 7B). The average size of Cu particles in Cu_14_–PET/CB after the reaction was 1.0 ± 0.2 nm, which was almost the same as that before the reaction (0.9 ± 0.2 nm). Conversely, the average size of Cu particles in Cu_14_–CHT/CB after the reaction was 1.4 ± 0.3 nm, 1.56 times larger than that before the reaction (0.9 ± 0.2 nm), indicating NC aggregation. These results are also consistent with the correlation of Cu─Cu bond intensity in the Cu K‐edge FT‐EXAFS spectra. Thus, the prolonged CO_2_RR results in the removal of all neutral ligands from Cu_14_–CHT NC, leading to aggregation, which significantly influences selectivity and stability (Figure 7C). These findings highlight the pivotal role of ligand stability in governing the structural integrity, electrocatalytic performance, and product selectivity of Cu NCs during CO_2_RRs.

## 3 Conclusion

In this study, we successfully synthesized Cu_14_ NCs coprotected with SR (CHT or PET) and PPh_3_ and investigated their catalytic effects in electrochemical CO_2_RRs. While the geometrical and electronic architectures of these NCs were comparable, we observed slight variations in their internal metallophilic interactions and intracluster interactions at the external ligand surfaces. These subtle differences significantly affected the selectivity of CO_2_RR products. Although the primary CO_2_RR products for both NCs were CO and HCOOH across a range of potentials, prolonged measurements revealed significant differences in the FEs (FE_HCOOH_ = 12% for Cu_14_–CHT and 35% for Cu_14_–PET). Our investigation revealed that the selectivity changes after the complete desorption of neutral ligands. Thus, the stability of the cluster and its ligand coordination are crucial factors in determining the selectivity and sustained performance of these NCs in electrochemical CO_2_RRs. Our results provide guidance for the development of highly selective and durable CO_2_RR catalysts through precise ligand engineering.

## 4 Experimental Section

### Synthesis of [Cu_14_(CHT)_3_(PPh_3_)_8_H_10_]^+^ and [Cu_14_(PET)_3_(PPh_3_)_8_H_10_]^+^

The synthesis was carried out in a 50 mL vial in an ice bath under atmospheric conditions (Scheme S1, Supporting Information for Cu_14_–CHT and Scheme S2, Supporting Information for Cu_14_–PET). First, commercially available tetrakis(acetonitrile)copper(I) tetrafluoroborate ((Cu(CH_3_CN)_4_BF_4_), 160 mg, 0.509 mmol) was dissolved by adding acetonitrile (4 mL) and chloroform (4 mL) in that order, followed by 22.8 µL of cyclohexanthiol (or 25 µL of phenylethanethiol in the case of Cu_14_–PET). Then, 130 mg of triphenylphosphine (PPh_3_) was added to the reaction mixture and stirred for 10 min. After fully dissolving the PPh_3_ powder in solution, 30 mg of NaBH_4_ (in 3 mL of methanol) was quickly added. The color of the solution immediately changed from clear to dark red. The reaction mixture was stirred for 5 h until the color of the solution changed from dark red to bright reddish brown. Subsequently, the solvent was removed using a rotary evaporator, and the residue was washed twice with 10 mL of methanol and dissolved in 10 mL of chloroform. Next, 10 mL of hexane was poured gently and layered on top of the chloroform solution. The mouth of the vial was sealed and allowed to stand in a refrigerator for 1 day to obtain yellow [Cu_14_(CHT)_3_(PPh_3_)_8_H_10_]^+^ (or [Cu_14_(CHT)_3_(PPh_3_)_8_H_10_]^+^) crystals (Figure S3A,B, Supporting Information).

## Conflict of Interest

The authors declare no conflict of interest.

## Author Contributions

Y.S. and M.O. contributed equally to this work. S.B., T.K., and Y.N. conceived the research and designed the experiments. S.B., S.H., T.K., and Y.N. designed the synthesis and electrocatalytic tests. Y.S., M.O., T.T., K.I., and K.T. performed the synthesis, characterization, and electrocatalytic activity procedures. S.B., S.H., Y.Y., and S.S. performed the structural analysis. T.K., S.K., and S.Y. performed the XAFS measurements. D.O. and G.M. performed the HAADF‐STEM and EES elemental mapping. S.B., T.K., and Y.N. wrote the manuscript.

## Supporting information



Supporting Information

## Data Availability

The data that support the findings of this study are available from the corresponding author upon reasonable request.
