# Supplementary material for: Ligand‐Dependent Intracluster Interactions in Electrochemical CO2 Reduction Using Cu14 Nanoclusters
Source: Small. 2024 Dec 4;21(16):2409910. doi: 10.1002/smll.202409910 (PMC12019909; doi:10.1002/smll.202409910)
Supplement: Supplementary file 1 — Supporting Information [file SMLL-21-2409910-s001.pdf]

NANO · MICRO small

Supporting Information

for *Small*, DOI 10.1002/smll.202409910

Ligand-Dependent Intracluster Interactions in Electrochemical CO_2 Reduction Using Cu_{14} Nanoclusters

Yamato Shingyouchi, Masaki Ogami, Sourav Biswas, Tomoya Tanaka, Maho Kamiyama, Kaoru Ikeda, Sakiat Hossain, Yusuke Yoshigoe, D. J. Osborn, Gregory F. Metha, Tokuhisa Kawawaki and Yuichi Negishi**

Supporting Information

Ligand-Dependent Intracluster Interactions in Electrochemical CO_2 Reduction Using Cu_{14} Nanoclusters

Author Information

Yamato Shingyouchi[1,†], *Masaki Ogami*[1,†], *Sourav Biswas*[2], *Tomoya Tanaka*[1], *Maho Kamiyama*[1], *Kaoru Ikeda*[1], *Sakiat Hossain*[2], *Yusuke Yoshigoe*[1], *D. J. Osborn*[3], *Gregory F. Metha*[3], *Tokuhisa Kawawaki*[1,2,4*], and *Yuichi Negishi*[4,5,*]

Affiliations

[1]Department of Applied Chemistry, Faculty of Science, Tokyo University of Science, Kagurazaka, Shinjuku-ku, Tokyo 162-8601, Japan

[2]Research Institute for Science and Technology, Tokyo University of Science, 2641 Yamazaki, Noda, Chiba 278-8510, Japan

[3]Department of Chemistry, University of Adelaide, Adelaide, South Australia, 5005, Australia

[4]Carbon Value Research Center, Tokyo University of Science, 2641 Yamazaki, Noda, Chiba 278-8510, Japan

[5]Institute of Multidisciplinary Research for Advanced Materials, Tohoku University, Katahira 2-1-1, Aoba-ku, Sendai 980-8577, Japan

†These authors contributed equally to this work.

*Corresponding author E-mail: kawawaki@rs.tus.ac.jp (T.K.), yuichi.negishi.a8@tohoku.ac.jp (Y.N.)

S1. Experimental Section

S1.1. Chemicals

All chemicals were commercially obtained and used without further purification.

Tetrakis(acetonitrile)copper(I) tetrafluoroborate [$Cu(CH_3CN)_4(BF_4)$], cyclohexanethiol, sodium borohydride ($NaBH_4$), and HPLC grade solvents-chloroform ($CHCl_3$) and methanol (MeOH) were purchased from Tokyo Chemical Industry Co., Ltd. Acetonitrile, triphenylphosphine (PPh_3), boron nitride (BN), barium sulfate ($BaSO_4$), copper (Cu) standard solution (1000 mg L^{-1}), bismuth standard solution (1000 mg L^{-1}), water, 2-propanol, Nafion®, deuterium oxide (D_2O) and dimethyl sulfoxide (DMSO) were procured from FUJIFILM Wako Pure Chemical Corporation. Hexane, nitric acid (HNO_3), hydrochloric acid (HCl) and potassium bicarbonate ($KHCO_3$) were procured from Kanto Chemical Co., Inc. 2-phenylethanethiol was obtained from Sigma-Aldrich Co. Carbon black (Vulcan XC-72) was procured from Fuel Cell Earth. Carbon paper (SIGRACET®; GDL 22BB) was procured from SGL carbon. Pure Milli-Q water (>18 MΩ × cm) was generated using a Merck Millipore Direct 3 UV system.

S1.2. Preparation of catalyst slurry

To prepare Cu_{14} NCs-loaded electrocatalysts (Cu_{14}–SR/CB), Cu_{14} NCs ($[Cu_{14}(CHT)_3(PPh_3)_8H_{10}]^+$ and $[Cu_{14}(PET)_3(PPh_3)_8H_{10}]^+$; PET = 2-phenylethanethiolate, CHT = cyclohexanethiolate) were loaded onto the CB using the impregnation method (Scheme S3). Specifically, Cu_{14} NCs crystal were dissolved in chloroform, and the concentration was measured by ICP-MS. Then the NC solution was added to the CB. In this process, the Cu loading ratio was set to 10 wt%. The mixture was mixed at room temperature until the solvent evaporated. Finally, Cu_{14}–SR/CB (SR = CHT and PET) were obtained by evacuating overnight in a desiccator.

S1.3. Electrochemical measurements

All electrochemical measurements for the CO_2 reduction reaction (CO_2RR) were performed with an ECstat-302 (EC FRONTIER, Japan) with a flow cell (EC FRONTIER, Japan). First, to prepare the catalyst slurry, Cu catalyst (12 mg) was added to a solution consisting of ultrapure water (2 mL), 2-propanol (0.5 mL), and Nafion® solution (10 μL). The obtained mixture was sonicated in an ice-water

bath for 30 min to disperse the Cu catalyst (Cu_{14}–SR/CB) and afford a catalyst slurry. Then, the catalyst slurry (1.1 mL) was sprayed on carbon paper (SIGRACET®GDL 22BB) (φ = 2 cm), which was used as the working electrode. A Pt mesh electrode was used as the counter electrode. A silver/silver chloride (Ag/AgCl) electrode was used as the reference electrode. Each electrode was set in an electrochemical measurement system containing 0.1 M $KHCO_3$ (pH = ≈7 under CO_2 sat.) as the electrolyte. In the measurements, CO_2 gas was bubbled for 15 min and then cyclic voltammetry (CV) was conducted for cleaning the electrodes. After CV, Chronoamperometry (CA) was performed under CO_2 (flow rate: 10 mL min^{-1}) at −0.6 V (vs. RHE) for 30 min. After that, the electrolyte is replaced and CO_2 bubbling is performed for the next CA. The detailed scheme is shown in Scheme S4. Gas products were analyzed by online gas chromatograph (Shimadzu, GC-8A, TCD or FID; Ar or N_2 carrier gas, respectively). Liquid products were analyzed by ^1H NMR spectroscopy. Typically, 500 μL of electrolyte after electrolysis was mixed with 99.5 μL of D_2O containing 0.05 μL of DMSO as internal standard.

S1.4. Characterization

The diffuse reflection spectra were acquired at ambient temperature with a V-670 spectrometer (JASCO, Tokyo, Japan). The wavelength-dependent optical data [$I(w)$] were converted to energy-dependent data [I(E)] with the following equation that conserved the integrated spectral areas: $I(E) = I(w)/|\partial E/\partial w| \propto I(w) \times w^2$.

ESI-MS was performed with a microTOF II reflectron time-of-flight mass spectrometer (Bruker, Massachusetts, USA). In these measurements, a NC solution with a concentration of ≈10 μg/mL in a mixture of chloroform and methanol was electrosprayed at a flow rate of 180 μL/h. Detailed parameters were the following; capacity, 3500 V; nebulizer, 2.0 Bar; dry gas, 3.0 L/min; dry temperature, 180 °C; ion energy, 1.0 eV; collision energy, 3.0 eV; transfer time, 240.0 μs.

ICP-MS was performed with an Agilent 7850c spectrometer (Agilent Technologies, Tokyo, Japan). Bi was used as the internal standard. Cu standard solution were used for drawing calibration line. The ICP-MS measurements were performed for the solution before mixing $[Cu_{14}(CHT)_3(PPh_3)_8H_{10}]^+$ and

$[Cu_{14}(PET)_3(PPh_3)_8H_{10}]^+$ with CB to estimate the adsorbed or loaded Cu content.

TEM images were recorded with a H-9500 electron microscope (HITACHI, Tokyo, Japan) or JEM-2100 electron microscope (JEOL, Tokyo, Japan) operating at 200 kV, typically using magnification of 600 000.

The high-angle annular dark field scanning TEM (HAADF-STEM) images were obtained by ultra-high-resolution transmission electron microscope (The FEI Titan Themis 80–200) operating at 200 kV, with a beam convergence semi angle of 25 mrad and HAADF collection angle from 56–200 mrad. Elemental maps were acquired using a super X detector and low background sample holder.

FT-IR spectra of the product were obtained using the attenuated total reflectance (ATR) method in the region between 400 and 4000 cm^{-1} by a FT/IR-4600-ATR-PRO ONE spectrometer (JASCO, Tokyo, Japan) equipped with a DLATGS detector as the average of 50 scans at 4 cm^{-1} resolution.

The X-ray photoelectron spectroscopy (XPS) spectra were collected by using a JPS-9010MC electron spectrometer (JEOL, Tokyo, Japan) at a base pressure of ~2×10^{-8} Torr. X-rays from the Mg-Kα line (1253.6 eV) were used for excitation. Each NCs was deposited on an Au plate and the spectra were calibrated with the peak energies of Au $4f_{7/2}$ (83.8 eV). Each catalyst was applied on the carbon tape and the spectra were calibrated with the peak energies of C 1s (284.6 eV).

X-ray absorption fine structure (XAFS) measurements were performed at beamline BL01B1 of the SPring-8 facility of the Japan Synchrotron Radiation Research Institute (proposal numbers 2022B1823, 2023A1675 and 2023B1825). The incident X-ray beam was monochromatized with a Si(111) double-crystal monochromator. Cu K-edges of XAFS spectra of all samples (as well as Cu foil, Cu_2O powder, and CuO powder as a reference) were recorded in transmission mode with ionization chambers. The X-ray energies for the Cu K-edges were calibrated with Cu foil, respectively. X-ray absorption near-edge structure (XANES) and extended XAFS (EXAFS) spectra were analysed with xTunes[1] as follows. The χ spectra were extracted by subtracting the atomic absorption background by cubic spline interpolation and normalized to the edge height. The normalized data were used as the XANES spectra. The k^3-weighted χ spectra in the k range 3.0–12.0

$Å^{-1}$ for the Cu K-edges were Fourier-transformed into *r* space for structural analysis.

S1.5 X-ray Crystallography

A single crystal was immersed in the cryoprotectant Parabar 10312 (Hampton Research, 34 Journey, Aliso Viejo, CA 92656-3317 USA) and kept at 90 K during diffraction data collection. A Bruker D8 QUEST diffractometer was used to collect the diffraction data for the single crystal using monochromated Mo Kα radiation (λ = 0.71073 Å). Although many crystals from different batches were checked for the diffraction experiment, all of them lacked higher angle data. However, the collected diffraction data was good enough to obtain a structure containing Cu(I) ions, S, P, and a few C atoms, which was solved by SHELXT[2] using the intrinsic phasing method in Apex3 Bruker Software Suite.[3] Later, during refinement the full crystal structure was completed using the full-matrix least squares method against F2 by SHELXL-2018/3 in Olex2 GUI[4] All the atoms including thiolates, triphenylphosphine and chloroform solvent were refined anisotropically. A few disordered phenyl rings were fixed by AFIX 66. Although few check cif alerts are there, we have provided a comprehensive response as follows.

start Validation Reply Form

_vrf_PLAT308_ALERT_2_A

Problem: Single Bonded Metal Atom in Structure (Unusual)

Response: This single bond Cu is associated with a bonding hydride coordinated with Cu(I) ions.

_vrf_PLAT910_ALERT_3_B

Problem: Missing # of FCF Reflection(s) Below Theta (Min).

Response: Some of the FCF reflections were below theta minimum due to obstruction from the beam stop because of long distance of lattice constant for Cu NCs.

S2. Additional Tables

Table S1. Crystal data and structure refinement parameters of $[Cu_{14}(CHT)_3(PPh_3)_8H_{10}]^+$.

Identification code	Cu14CHT _90K
Empirical formula	$C_{163.25}H_{154.25}Cl_4Cu_{14}P_8S_3$
CCDC number	2371368
Formula weight	3491.41
Temperature/K	90.15
Crystal system	triclinic
Space group	*P*-1
a/Å	17.5724(7)
b/Å	17.7419(7)
c/Å	32.9669(14)
α/°	79.7540(10)
β/°	75.1940(10)
γ/°	76.6830(10)
Volume/Å^3	9591.9(7)
Z	2
ρ_{calc}/g cm^{-3}	1.209
μ/mm^{-1}	1.711
F(000)	3552.0
Crystal size/mm^3	0.534 × 0.363 × 0.144
Radiation	MoKα (λ = 0.71073)
2Θ range for data collection/°	4.022 to 62.638
Index ranges	$-25 \leq h \leq 25, -23 \leq k \leq 23, -46 \leq l \leq 42$
Reflections collected	126716
Independent reflections	47185 [R_{int} = 0.0439, R_{sigma} = 0.0581]
Data/restraints/parameters	47185/472/1868
Goodness-of-fit on F^2	1.057
Final R indexes [I>=2σ (I)]	R_1 = 0.0656, wR_2 = 0.1736
Final R indexes [all data]	R_1 = 0.0960, wR_2 = 0.1946
Largest diff. peak/hole / e Å^{-3}	1.99/-1.49

Table S2. Crystal data and structure refinement parameters of $[Cu_{14}(PET)_3(PPh_3)_8H_{10}]^+$.

Identification code	Cu14PET_90K
Empirical formula	$C_{168}H_{147}Cu_{14}P_8S_3$
CCDC number	2371369
Formula weight	3399.35
Temperature/K	90.15
Crystal system	triclinic
Space group	*P*-1
a/Å	17.2456(17)
b/Å	24.530(3)
c/Å	24.558(3)
α/°	74.744(3)
β/°	74.111(3)
γ/°	72.536(3)
Volume/Å^3	9344.4(16)
Z	2
ρ_{calc}/g cm^{-3}	1.208
μ/mm^{-1}	1.700
F(000)	3458.0
Crystal size/mm^3	0.12 × 0.094 × 0.082
Radiation	MoKα (λ = 0.71073)
2Θ range for data collection/°	3.77 to 51.362
Index ranges	$-21 \le h \le 21$, $-29 \le k \le 29$, $-29 \le l \le 29$
Reflections collected	107581
Independent reflections	35136 [R_{int} = 0.1583, R_{sigma} = 0.1910]
Data/restraints/parameters	35136/90/1414
Goodness-of-fit on F^2	0.980
Final R indexes [I>=2σ (I)]	R_1 = 0.0821, wR_2 = 0.1981
Final R indexes [all data]	R_1 = 0.1606, wR_2 = 0.2380
Largest diff. peak/hole / e Å^{-3}	0.75/-0.84

S3. Additional Schemes

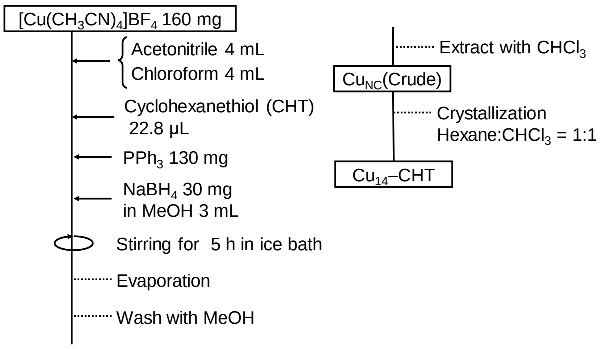

Scheme S1. Schematic of the synthesis protocol for $[Cu_{14}(CHT)_3(PPh_3)_8H_{10}]^+$.

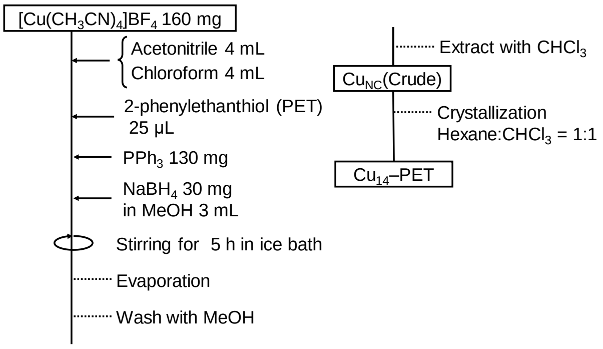

Scheme S2. Schematic of the synthesis protocol for $[Cu_{14}(PET)_3(PPh_3)_8H_{10}]^+$.

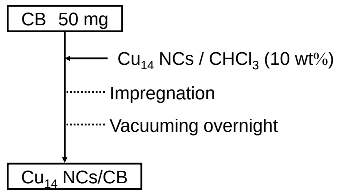

Scheme S3. Schematic of the preparation protocol for Cu_{14}–SR/CB (SR = CHT and PET).

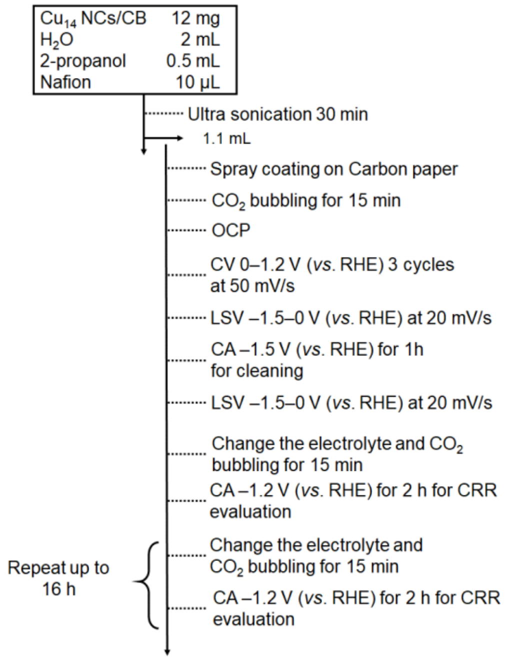

Scheme S4. Schematic of the measurement protocol for electrochemical CO_2RR.

S4. Additional Figures

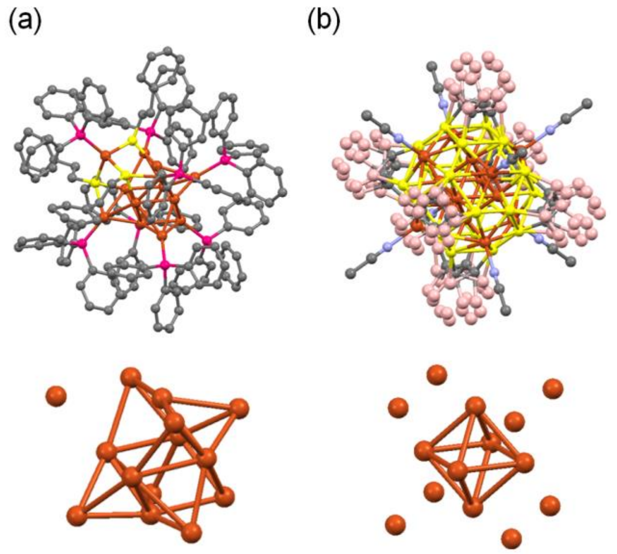

Figure S1. The total and core geometric structure of Cu_{14} NCs ((a) $[Cu_{14}(PET)_3(PPh_3)_8H_{10}]^+$ and (b) $Cu_{14}(C_2B_{10}H_{10}S_2)_6(CH_3CN)_8$). All the carbon parts are removed from the ligands, anionic part and the solvent part also removed for the clarity. Color legend: orange = Cu; yellow = S; magenta = P; dark grey = C; blue = N; pink =B.

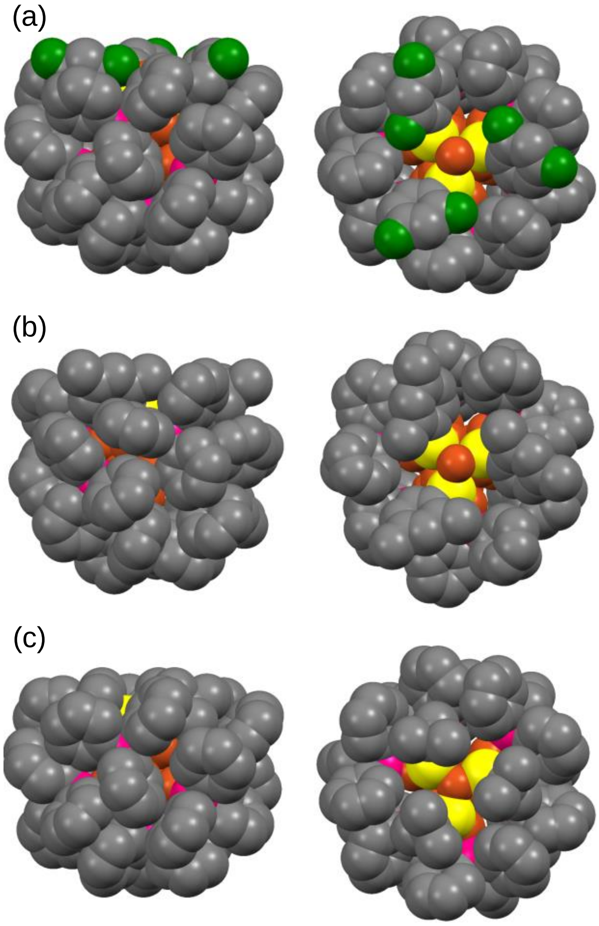

Figure S2. The geometric structure of Cu_{14} NCs ((a) $[Cu_{14}(SC_6H_3F_2)_3(PPh_3)_8H_{10}]^+$, (b) $[Cu_{14}(SPhMe_2)_3(PPh_3)_8H_{10}]^+$ and (c) $[Cu_{14}(^tBuS)_3(PPh_3)_7H_{10}]^+$). All the carbon parts are removed from the ligands, anionic part and the solvent part also removed for the clarity. Color legend: orange = Cu; yellow = S; magenta = P; dark grey = C; green = F. In (a) and (b), PPh_3 at outstanding Cu site is omitted for clarity.

(a) (b)

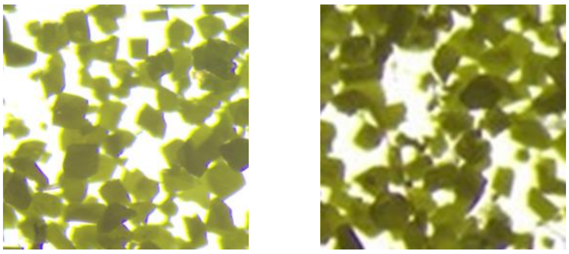

Figure S3. Photographs of single crystals for (a) $[Cu_{14}(CHT)_3(PPh_3)_8H_{10}]^+$ and (b) $[Cu_{14}(PET)_3(PPh_3)_8H_{10}]^+$.

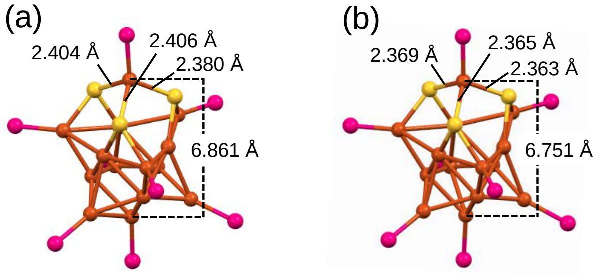

Figure S4. The Cu–S bond lengths in $Cu(SR)_3(PPh_3)$ motif and total distance between two opposite Cu atoms for (a) Cu_{14}–CHT and (b) Cu_{14}–PET. All of carbon and hydrogen is removed for the clarity. Color legend: orange = Cu; yellow = S; magenta = P.

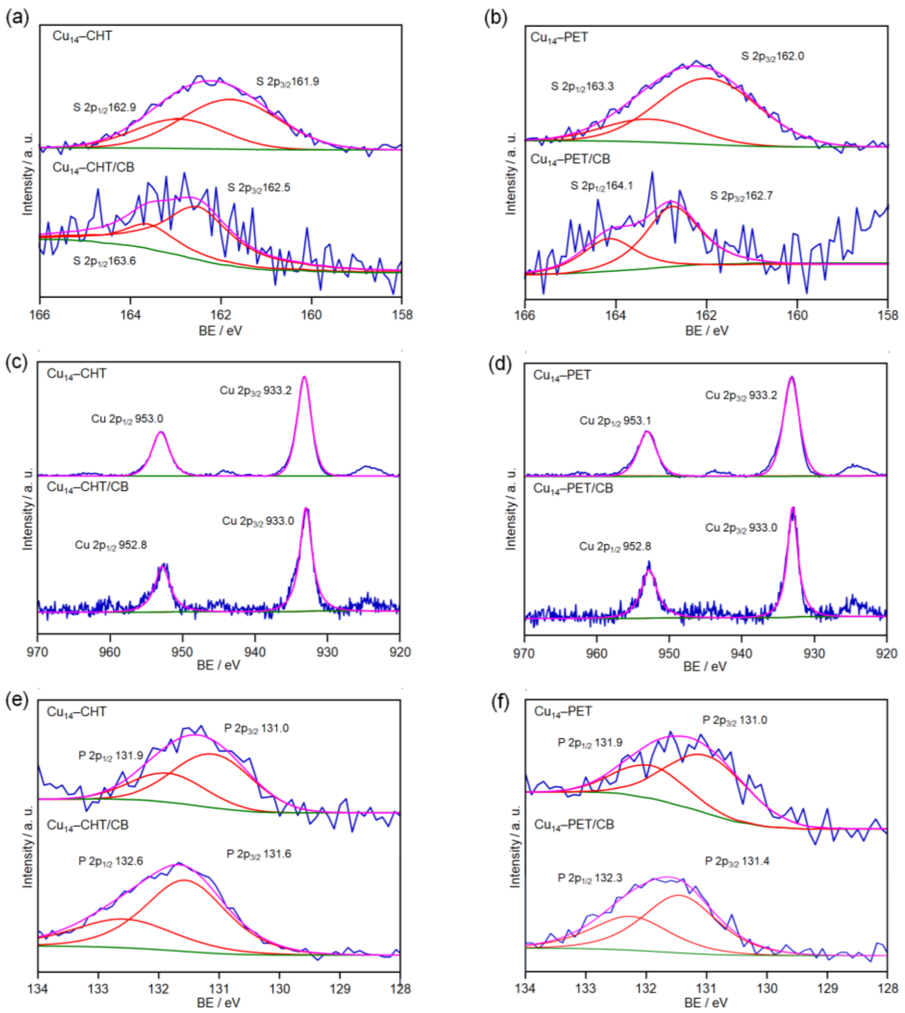

Figure S5. (a, b) S 2p, (c, d) Cu 2p and (e, f) P 2p XPS spectra (blue line) and their fitting results (red, green, and magenta lines) for (a, c, e) Cu_{14}–CHT and (b, d, f) Cu_{14}–PET before and after adsorption on CB. In (a, b, e, f), the slight shifts in the XPS peaks at S 2p (≈0.6 eV) and P 2p (≈0.5 eV) before and after deposition on CB, might be due to slight changes of the geometry of Cu_{14}–SR. The peak at ≈155 eV is attributed from Si 2s which is contaminated from agate mortar (SiO_2) in preparation process of samples. Since most of the X-rays were absorbed by the CB, which is a porous support with a large specific surface area, the noise was very large in the sample supported on CB (Cu_{14}–SR/CB).

(a)

(b)

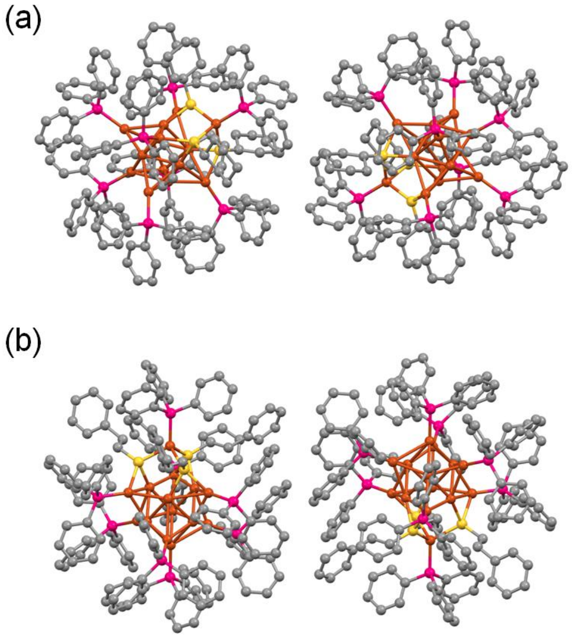

Figure S6. The expanded unit cell structure (a) Cu_{14}−CHT and (b) Cu_{14}−PET architecture. All of hydrogen is removed for the clarity. Color legend: orange = Cu; yellow = S; magenta = P; dark grey = C.

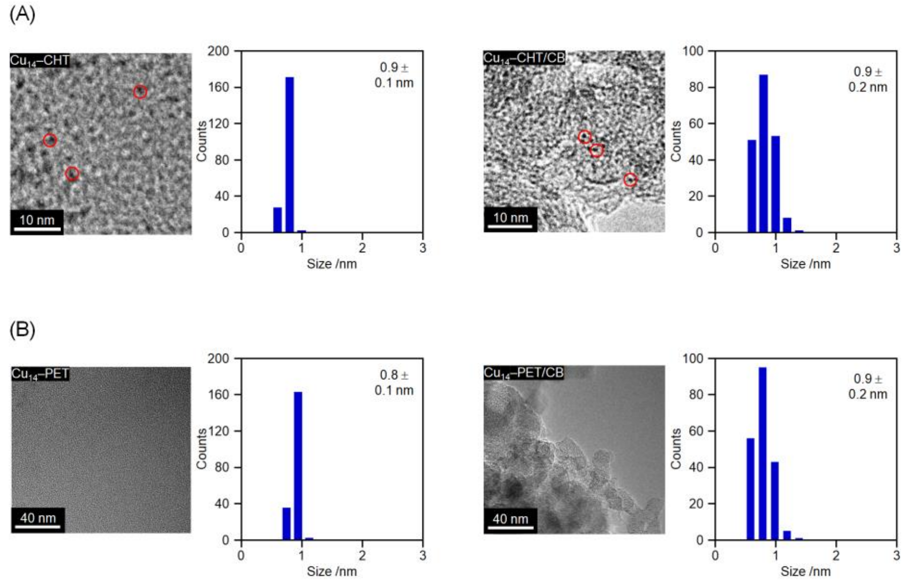

Figure S7. (A) TEM images and resulting histograms of the particle-size distribution for Cu_{14}–CHT and Cu_{14}–CHT/CB. (B) TEM images with lower magnification and a wider field of view for Cu_{14}–PET and Cu_{14}–PET/CB.

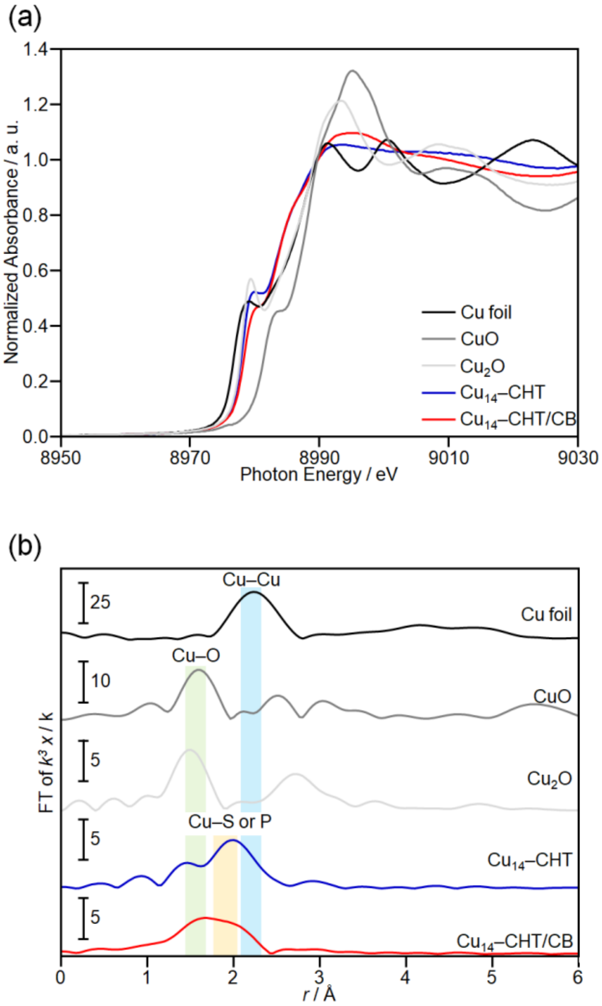

Figure S8. (a) Cu K-edge XANES spectra and (b) FT-EXAFS spectra for Cu_{14}−CHT and Cu_{14}−CHT/CB. In (a, b), Cu K-edge XANES and FT-EXAFS spectra of Cu foil, CuO and Cu_2O powder are also shown for comparison. In (b), the peak at ≈1.6, ≈1.8 and 2.0−2.6 Å are assigned to the Cu−C or O, Cu−S or P, and Cu−Cu bond, respectively. The sample of Cu_{14}−CHT was measured at 10 K.

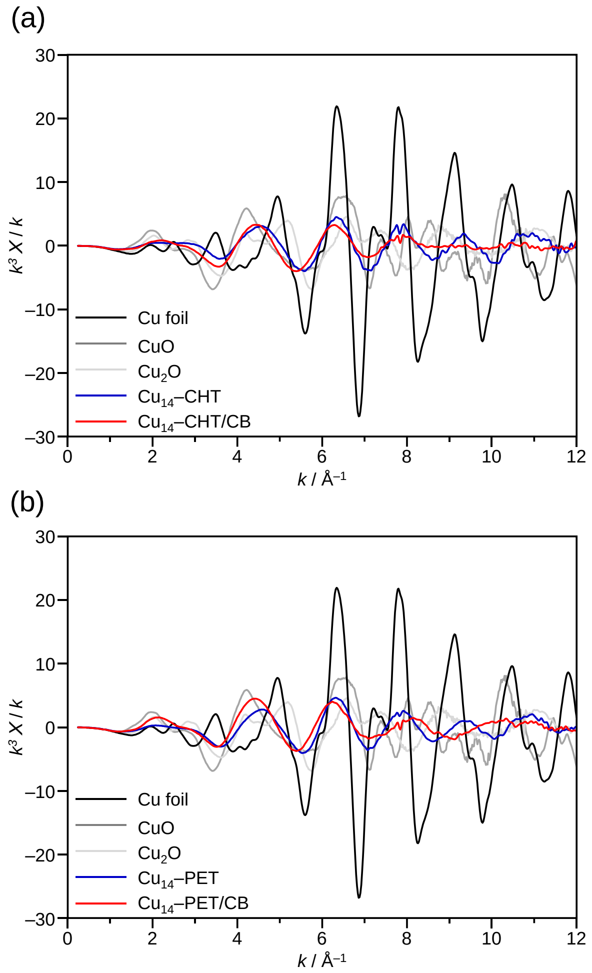

Figure S9. Results of Cu K-edge EXAFS spectra for $[Cu_{14}(CHT)_3(PPh_3)_8H_{10}]^+$ and $[Cu_{14}(PET)_3(PPh_3)_8H_{10}]^+$, and their catalysts. The EXAFS spectra of Cu foil, CuO powder and Cu_2O powder are also shown for comparison. The sample of Cu_{14}–CHT and Cu_{14}–PET were measured at 10 K.

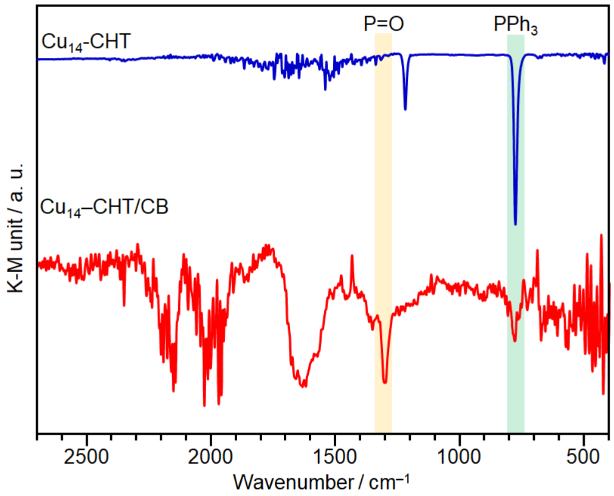

Figure S10. FT-IR spectra for Cu_{14}–CHT and Cu_{14}–CHT/CB.

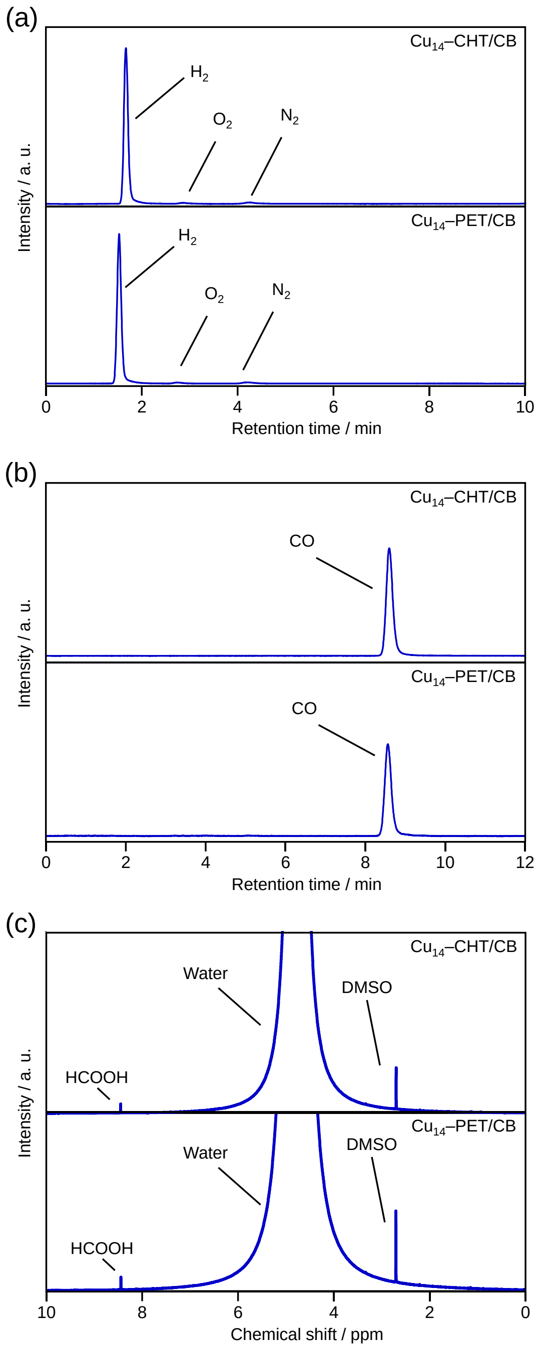

Figure S11. The raw data for electrocatalytic CO_2RR results. (a) GC-TCD, (b) GC-FID and (c) ^{1}H NMR spectra after applied potential at −1.2 V vs. RHE for 1 h in 0.1 M $KHCO_3$ aq. under CO_2 flow on Cu_{14}–SR/CB (SR = CHT and PET). In (c), Water and DMSO were observed from an electrolyte and internal standard, respectively.

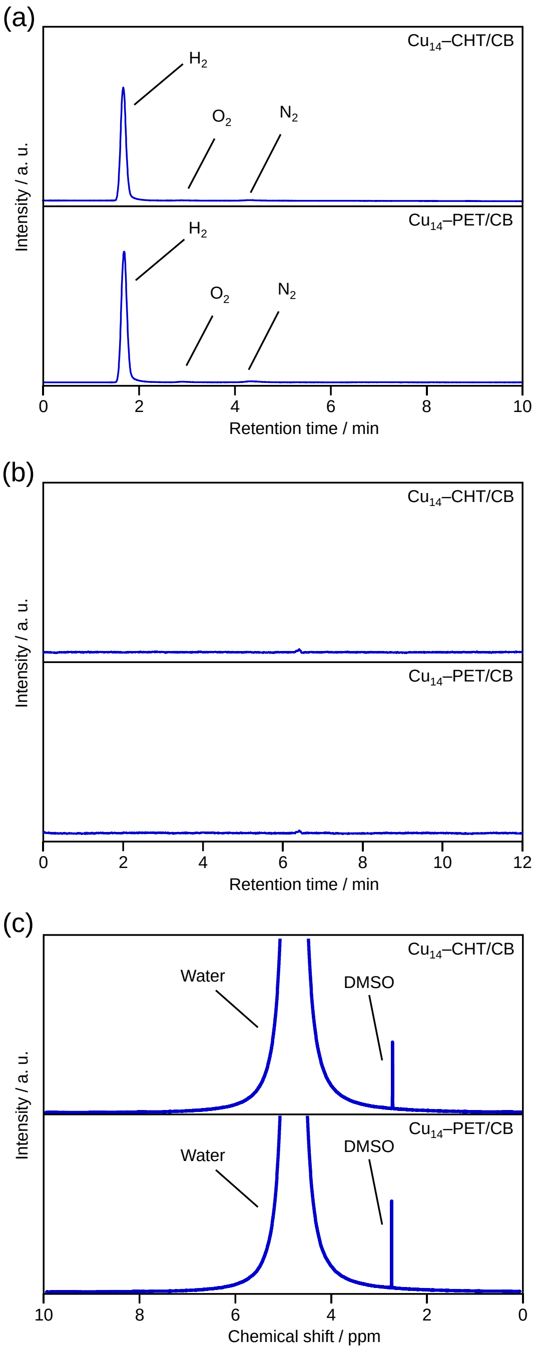

Figure S12. The raw data for electrocatalytic CO_2RR results. (a) GC-TCD, (b) GC-FID and (c) ^1H NMR spectra after applied potential at −1.2 V vs. RHE for 1 h in 0.1 M $KHCO_3$ aq. under Ar flow on Cu_{14}–SR/CB (SR = CHT and PET). In (c), Water and DMSO were observed from an electrolyte and internal standard, respectively.

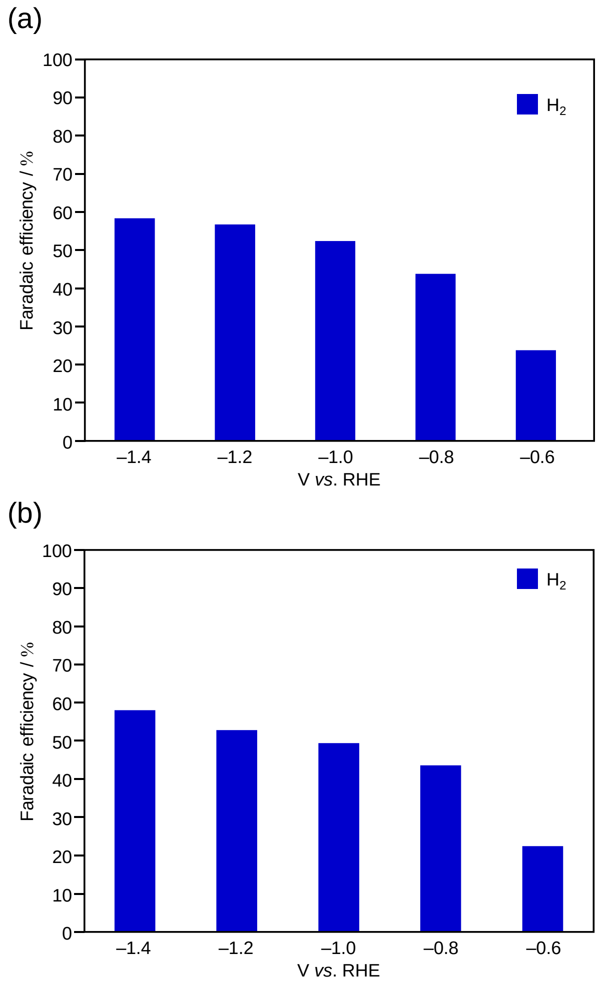

Figure S13. Electrocatalytic performances for CO_2RR in 0.1 M $KHCO_3$ aq. using gas-flow H-type cell. FE for (a) Cu_{14}–CHT/CB and (b) Cu_{14}–PET/CB under Ar flow.

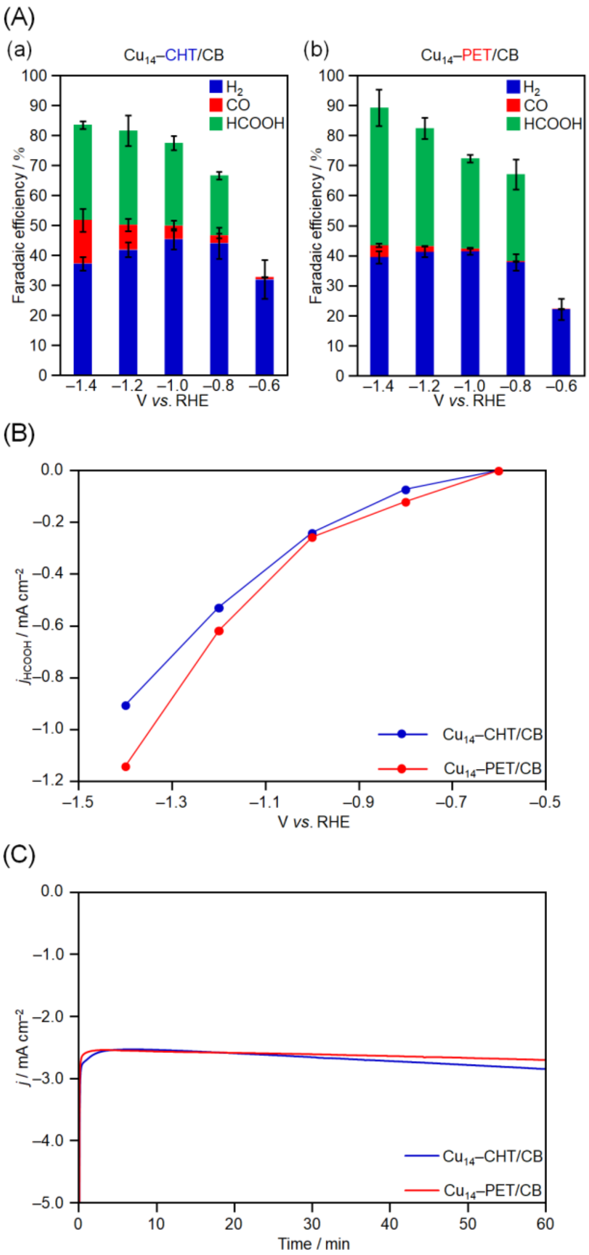

Figure S14. (A) FE for CO_2 reduction products and (B) the HCOOH partial current densities (j_{HCOOH}) at different applied potentials and (C) I-T curves of (a) Cu_{14}–CHT/CB and (b) Cu_{14}–PET/CB.

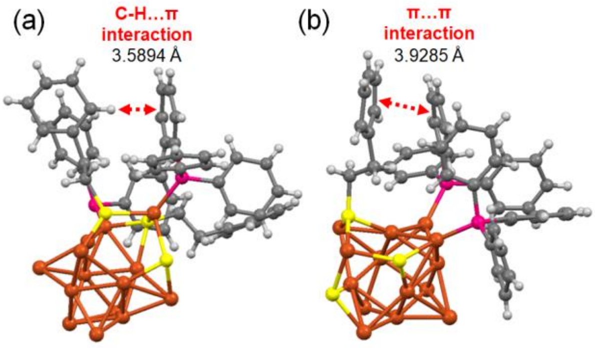

Figure S15. Relationship for (a) C−H···π and (b) π···π interaction in $[Cu_{14}(PET)_3(PPh_3)_8H_{10}]^+$. Some of the carbon, hydrogen, phosphine is removed for the clarity. Color legend: orange = Cu; yellow = S; magenta = P; dark grey = C; grey=H.

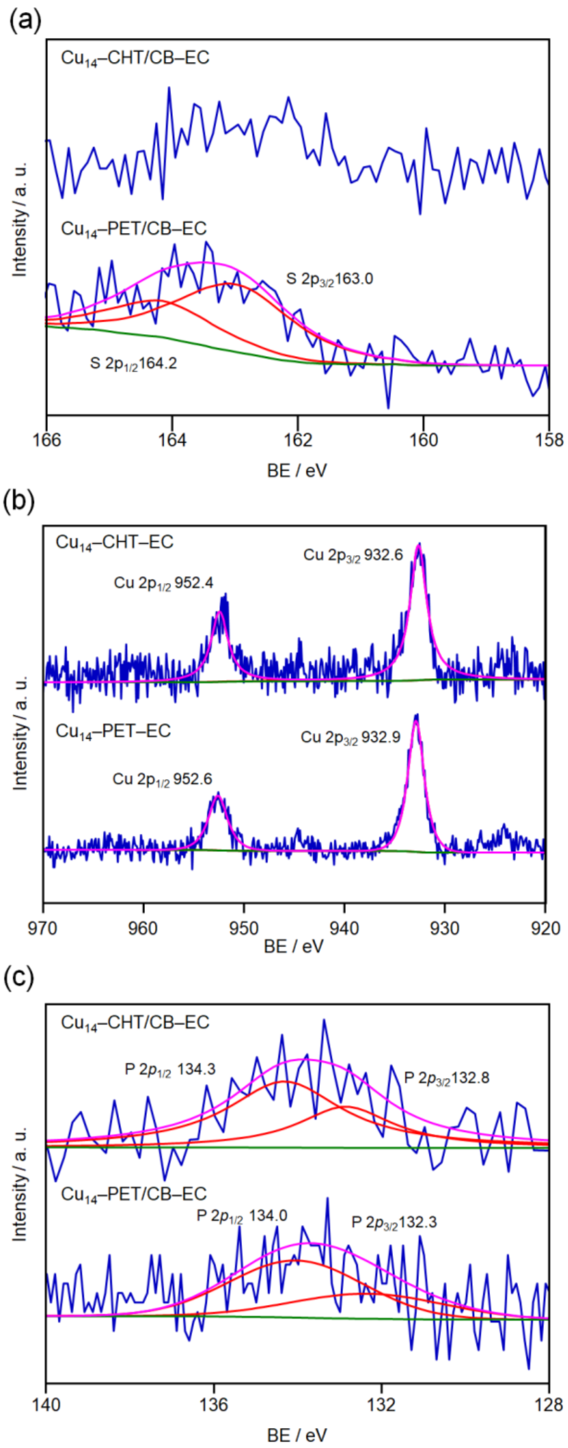

Figure S16. Results of (a) S 2p and (b) Cu 2p and (c) P 2p XPS spectra (blue line) and their fitting results (red, green, and magenta lines) for Cu_{14}–SR/CB-EC. The weak signals of P were observed in Cu_{14}–SR/CB-EC. This is presumably because the triphenylphosphine ligands on the Cu_{14}–SR moved to the CB as phosphine oxides, which were then dissolved in the electrolyte.

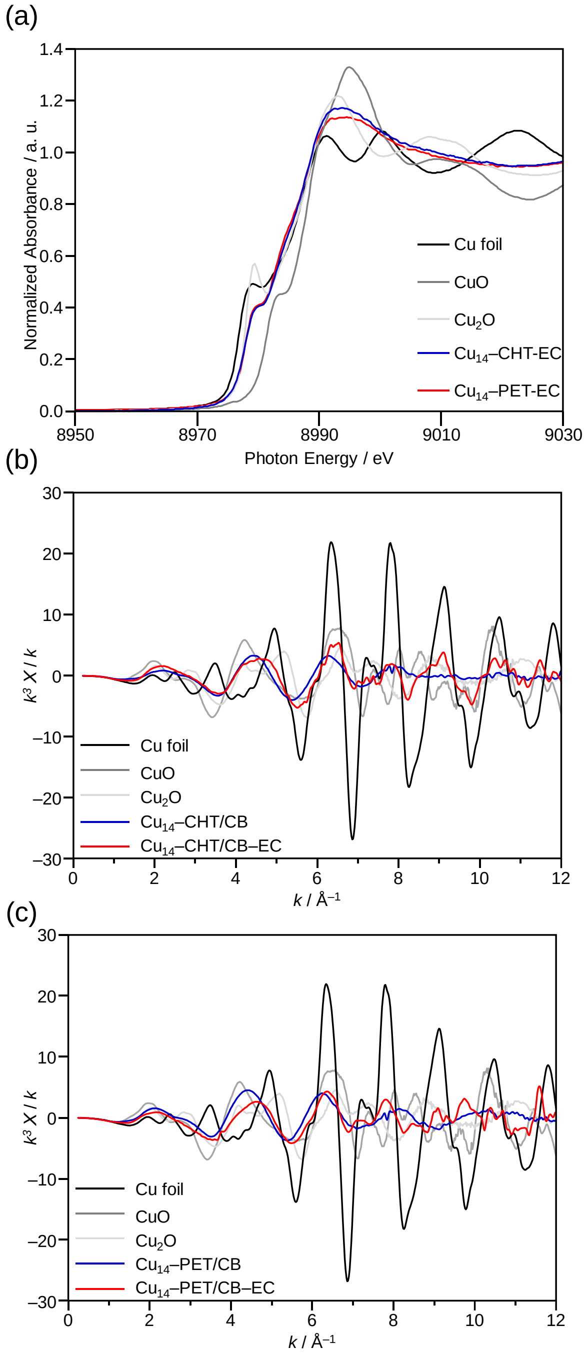

Figure S17. Results of Cu K-edge (a) XANES and (b, c) EXAFS spectra for Cu_{14}−SR/CB and Cu_{14}−SR/CB-EC. The EXAFS spectra of Cu foil, CuO powder and Cu_2O powder are also shown for comparison.

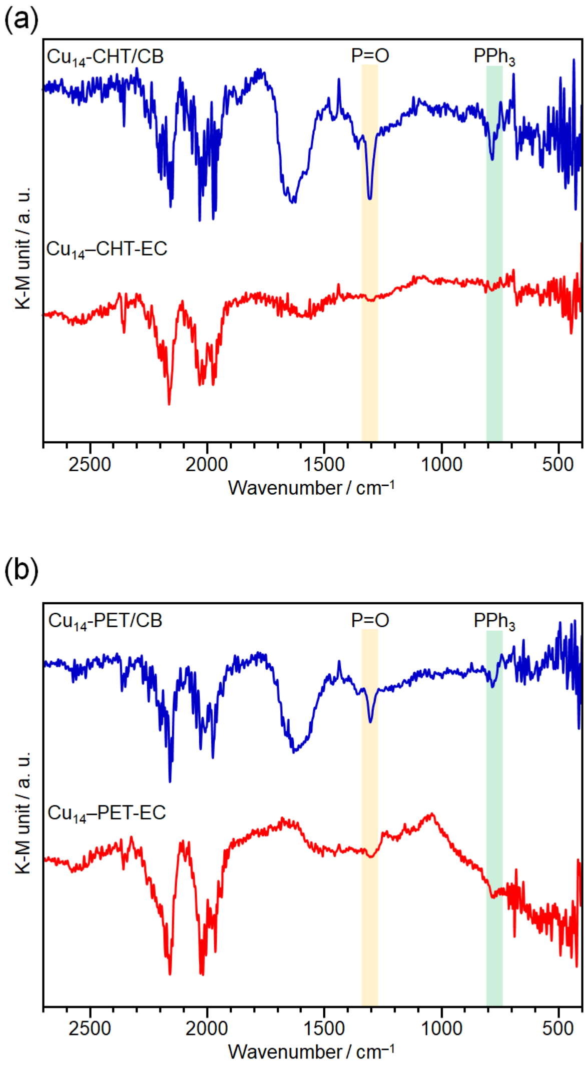

Figure S18. FT-IR spectra of $Cu_{14}(SR)_3(PPh_3)_8H_{10}$ (SR = CHT and PET)-loaded CB catalysts before and after electrocatalytic CO_2RR.

S5. References

[1] H. Asakura, S. Yamazoe, T. Misumi, A. Fujita, T. Tsukuda, T. Tanaka, *Radiat. Phys. Chem.* **2020**, *175*, 108270.

[2] G. M. Sheldrick, *Acta Crystallogr., Sect. A: Found. Adv.* **2015**, *71*, 3.

[3] Bruker APEX4 (v2021.10–0), Bruker AXS Inc., Madison, WI, USA, **2021**.

[4] a) G. M. Sheldrick, *Acta Crystallogr., Sect. C: Struct. Chem.* **2015**, *71*, 3; b) O. V. Dolomanov, L. J. Bourhis, R. J. Gildea, J. A. K. Howard and H. Puschmann, *J. Appl. Crystallogr.* **2009**, *42*, 339.
